# Clinical research and networks – a good marriage?

**DOI:** 10.1186/1753-6561-5-S1-L9

**Published:** 2011-01-10

**Authors:** Jeremy J Farrar

**Affiliations:** 1 Oxford University Clinical Research Unit, The Hospital for Tropical Diseases, Ho Chi Minh City, Vietnam

## 

The largest, most devastating outbreak of an infectious disease in modern history occurred in 1918, when a highly virulent influenza A H1N1 virus spread throughout the world and killed between 20 and 40 million people. This haunting memory has led to continued concern about the ongoing circulation of avian H5N1 influenza virus and sensitized the world to the potential of the recent H1N1 2009 pandemic. Considering the potential death toll of a 1918-like influenza pandemic, the still evolving HIV/AIDS epidemic, the spread of drug resistance, and the massive and unprecedented changes in the environment and societies throughout much of the developing world such collective global but more importantly regional investment must be a top priority. Investment in scientific and health care infrastructure and consideration of new paradigms for clinical science are required to address the global health challenges of the 21^st^ Century.

International cooperation, collaboration and sharing of data is essential, but this will only happen if there is trust engendered between scientists by long term shared endeavour via an equitable scientific partnership between the north and the south. Such partnerships cannot be generated quickly and only when the rich world feels threatened by something that might potentially spread to them from the developing world. The information from such research and the benefits need to be shared and flow equitably in both directions. There has been a remarkable explosion in the molecular and other basic sciences over the last twenty years. There is a very real danger that as we continue to neglect (and make increasingly and unnecessarily bureaucratic and complicated) patient orientated research and public health that it will be these areas that will hold back the phenomenal opportunities that might accrue from the basic scientific revolution. From SNPs to cellular responses, from cytokines to arrays and through to proteomics and beyond we can now deliver a mass of scientific data in minutes. Unless we can rationalise that data and put it into the context of a human being, the environment and community in which they live we will not deliver the promised benefits of this remarkable scientific age to people who need it most. We have neglected the clinical and public health bit of clinical science for too long and we have failed to build sufficient long term equitable scientific partnerships between the north and the south.

There is now a great opportunity to reinvigorate health research fully integrated with the basic and social sciences and to build strong international collaborations with the centre of gravity firmly based where the need is greatest. The potential opportunities that can arise from such collaborations and networks are enormous but like marriage such endeavours need a great deal of work in order to be successful and they may not be the answer to everything!

